# Recurrence Patterns and Overtreatment in Pure DCIS: A Retrospective Clinical and Radiological Follow-Up Study

**DOI:** 10.3390/jpm16060281

**Published:** 2026-05-25

**Authors:** Maria Concetta Torrione, Andrea Gaia Azzarito, Vanessa Marisi, Maria Francesca Savina, Angela Di Credico, Riccardo Luberti, Marzia Muzi, Claudia D’Eramo, Massimo Caulo, Andrea Delli Pizzi

**Affiliations:** 1 Unit of Radiology, “Santissima Annunziata” Hospital, 66100 Chieti, Italy; concettator@yahoo.it (M.C.T.); andreagaiaazzarito@gmail.com (A.G.A.); savina.mariafrancesca@outlook.it (M.F.S.); angeladicredico@gmail.com (A.D.C.); riccardoluberti1@gmail.com (R.L.); massimo.caulo@unich.it (M.C.); 2 Unit of Radiology, “San Timoteo” Hospital, 86039 Termoli, Italy; vanessamarisi@hotmail.it; 3 Breast Unit, “Gaetano Bernabeo” Hospital, 66026 Ortona, Italy; marziamuzi@gmail.com (M.M.); claudiaderamo@libero.it (C.D.); 4 ITAB—Institute for Advanced Biomedical Technologies,” G. d’Annunzio” University, 66100 Chieti, Italy; 5 Department of Neuroscience, Imaging and Clinical Sciences, “G. d’Annunzio” University, 66100 Chieti, Italy; 6 Department of Innovative Technologies in Medicine & Dentistry, “G. d’Annunzio” University, 66100 Chieti, Italy

**Keywords:** DCIS, local recurrence, breast cancer, radiotherapy, follow-up, overtreatment

## Abstract

**Background/Objectives:** The clinical management of ductal carcinoma in situ (DCIS) remains controversial due to its heterogeneous biological behavior and uncertain risk of progression. Standard treatment often includes surgery and radiotherapy, although the actual recurrence risk varies considerably among patients. This study aimed to evaluate recurrence patterns and associated clinicopathological factors in a large single-center cohort of patients with pure DCIS. **Methods:** We retrospectively analyzed 403 patients with histologically confirmed pure DCIS treated with breast-conserving surgery or mastectomy between 2016 and 2023. Clinical, imaging, pathological, and treatment-related variables were assessed. Descriptive and exploratory comparative analyses were performed between patients with and without ipsilateral recurrence. **Results:** A total of 417 lesions were analyzed, with 21 ipsilateral recurrences (5%) observed during follow-up. Among recurrent cases, 57% were non-invasive recurrent DCIS and 38% were invasive carcinomas. Most recurrences occurred in patients treated with breast-conserving surgery, and 52% of recurrent patients had not received adjuvant radiotherapy. All recurrent cases were estrogen receptor–positive at initial diagnosis, whereas none had received endocrine therapy. No clear association between recurrence patterns and tumor grade or tumor size emerged in this exploratory analysis. No distant metastases or disease-related deaths were observed during follow-up. **Conclusions:** Recurrence after treatment for pure DCIS was relatively uncommon and frequently non-invasive. Traditional clinicopathological variables alone appeared insufficient to consistently identify recurrence patterns in this cohort. These findings support the need for more individualized risk stratification approaches integrating clinical, imaging, and molecular factors in order to reduce potential overtreatment in selected patients with DCIS.

## 1. Introduction

Ductal carcinoma in situ (DCIS) is a non-invasive neoplastic proliferation of luminal epithelial cells confined to the ductolobular system of the breast, without invasion of the basement membrane [1,2]. Its incidence has increased markedly with the widespread adoption of screening mammography, currently accounting for approximately 20–25% of all screen-detected breast cancers and up to 85% of asymptomatic lesions [3,4]. DCIS most commonly presents as suspicious microcalcifications on mammography, while palpable masses are uncommon, occurring in less than 2% of cases [5].

Despite its non-invasive definition, DCIS demonstrates heterogeneous biological behavior. While some lesions may progress to invasive carcinoma, a substantial proportion are believed to remain indolent throughout a patient’s lifetime [6,7]. Autopsy and modeling studies suggest that up to 60–80% of DCIS lesions may never evolve into invasive disease, raising major concerns regarding overdiagnosis and overtreatment [8]. Consequently, there is growing interest in de-escalation strategies, including active surveillance trials for carefully selected low-risk patients [9,10,11]. Recent reviews and imaging-focused studies have further highlighted the evolving role of radiological follow-up and personalized risk assessment in this setting [12,13]. However, predicting which lesions will progress remains challenging, limiting widespread adoption of conservative management.

Standard treatment options for DCIS include breast-conserving surgery (BCS) or mastectomy, often followed by adjuvant radiotherapy (RT), and in selected cases, endocrine therapy. Multiple randomized trials have demonstrated that RT significantly reduces the risk of ipsilateral breast tumor recurrence, although it does not improve overall survival [12,14,15,16]. After mastectomy, RT is generally not indicated in the absence of invasive components or high-risk pathological features [17]. Overall, long-term breast cancer–specific survival exceeds 98% at 10 years, underscoring the excellent prognosis of DCIS regardless of treatment modality [7,18].

Accurate risk stratification remains a critical unmet need. Traditional clinicopathological factors—such as age, tumor size, histological grade, margin status, and presence of necrosis—have been variably associated with recurrence risk, but none have demonstrated sufficient predictive accuracy to guide individualized treatment reliably [19,20,21]. As a result, many patients continue to undergo aggressive treatments, including mastectomy, with uncertain incremental benefit.

In this retrospective single-center study, we analyzed a large consecutive cohort of patients with histologically confirmed pure DCIS treated over a 10-year period. The primary aim was to evaluate local recurrence during radiological follow-up, with a focus on tumor grade, radiotherapy, and other clinicopathological variables, in order to explore the potential for more personalized and less radical management strategies in selected patients.

## 2. Materials and Methods

This study received formal approval from the Ethical Committee of the University G. d’Annunzio of Chieti-Pescara, Italy; informed consent was waived by the same ethics committee that approved the study (Comitato Etico per la Ricerca Biomedica delle Province di Chieti e Pescara e dell’Università degli Studi “G. d’Annunzio” di Chieti e Pescara). We retrospectively reviewed 3771 breast biopsies performed at our Breast Unit between January 2016 and December 2023, including 938 stereotactic-guided vacuum-assisted biopsies and 2833 ultrasound-guided core biopsies. From these, we selected only cases with a final histopathological diagnosis of pure DCIS, defined as ductal carcinoma in situ without evidence of microinvasion, lobular neoplasia, or associated invasive carcinoma, in accordance with World Health Organization criteria [22].

Among initially eligible patients with biopsy-proven DCIS, cases showing microinvasion or invasive carcinoma on final surgical pathology were excluded from the final analysis, along with patients with lobular carcinoma in situ (*n* = 19), Paget disease (*n* = 2), a recent history of invasive breast cancer (*n* = 50), and contralateral recurrence during follow-up (*n* = 32) [19]. The final study cohort included 417 lesions in 403 patients with histologically confirmed pure DCIS treated with breast-conserving surgery or mastectomy and followed radiologically for at least six months.

All patients underwent preoperative bilateral mammography and breast ultrasound as standard of care. Breast MRI was selectively performed based on breast density, patient age, and the extent of microcalcifications, in line with current imaging recommendations for DCIS [23]. Imaging findings were classified according to the BI-RADS lexicon and were reviewed in consensus by a breast-dedicated radiologist and a resident in breast imaging. Mammographic features were categorized as microcalcifications, masses, or architectural distortion, while ultrasound reports documented lesion morphology and maximum diameter. Imaging evaluation in this study was performed primarily in the context of radiological follow-up and lesion presentation at diagnosis, rather than through advanced quantitative imaging analysis or radiomics-based approaches.

Histopathological variables included tumor grade, presence of necrosis, architectural subtype, hormone receptor status (ER,), and DIN classification. Treatment allocation, including the decision to administer adjuvant radiotherapy or endocrine therapy following breast-conserving surgery, reflected multidisciplinary clinical decision-making based on tumor characteristics, patient age, comorbidities, margin status, and patient preference, in accordance with contemporary institutional practice and evolving guidelines over the study period. Margin status and sentinel lymph node biopsy results were recorded. Sentinel lymph node biopsy was performed selectively, in line with current evidence suggesting that routine axillary staging is not indicated in pure DCIS [24]. Tumor staging followed the AJCC TNM system [25].

### Statistical Analysis

Descriptive statistics were used to summarize clinical, imaging, and pathological characteristics of the study population. Continuous variables were reported as mean ± standard deviation or median and range, as appropriate, whereas categorical variables were expressed as frequencies and percentages. Comparisons between patients with and without ipsilateral recurrence were performed using the chi-square test or Fisher’s exact test for categorical variables and Student’s *t*-test or Mann–Whitney U test for continuous variables, according to data distribution. All statistical analyses were considered exploratory. A *p*-value < 0.05 was considered statistically significant. Statistical analyses were performed using SPSS version 27 (IBM Corp., Armonk, NY, USA).

## 3. Results

A total of 417 pure DCIS lesions from 403 patients were included in the final analysis. Some patients presented with synchronous bilateral or multifocal lesions, resulting in a higher number of lesions than individual patients. Recurrence analyses were performed at the patient level. The mean age at diagnosis for the overall cohort was 56.7 ± 10.2 years.

Among all lesions, 75 (18%) were classified as low-grade (DIN1), 246 (59%) as intermediate-grade (DIN2), and 96 (23%) as high-grade (DIN3) DCIS. Treatment distribution included mastectomy in 28 cases, breast-conserving surgery (BCS) alone in 162 cases, BCS followed by radiotherapy (RT) in 185 cases, and BCS combined with RT and endocrine therapy in 16 cases. The clinicopathological characteristics of the overall cohort are summarized in Table 1.

**Figure 1:**
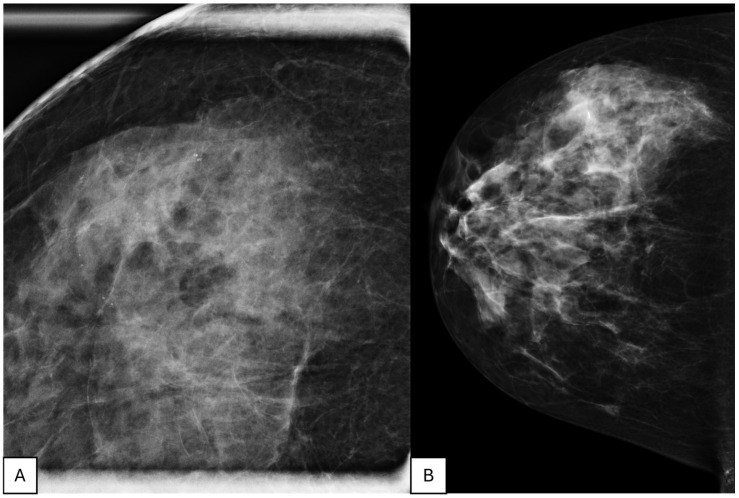
Radiological features and post-operative follow-up in a 43-year-old patient with Grade 1 DCIS. (**A**) Magnification mammographic view showing pathological microcalcifications at diagnosis, subsequently subjected to vacuum-assisted breast biopsy (VABB). (**B**) Post-operative mammogram showing the clip at the surgical site; the patient underwent breast-conserving surgery and radiotherapy.

Ipsilateral breast cancer recurrence was documented in 21 women (5%), consistent with recurrence rates reported in contemporary series [14,26]. Of these recurrences, 12 (57%) were non-invasive recurrent DCIS, 8 (38%) were invasive carcinomas, and 1 (5%) involved lymph node recurrence. No distant metastases were observed during follow-up [7,27]. The median age at recurrence was 57 years (range 43–73), and the median time to recurrence was 42 months (range 6–75 months). The pathological size of the initial DCIS ranged from 3 mm to 80 mm, with a mean size of 16 ± 11.5 mm. A representative case of Grade 1 DCIS with post-operative follow-up is shown in Figure 1. Regarding histological grade, 5 recurrent cases (24%) were initially classified as low-grade DCIS, 13 (62%) as intermediate-grade, and 3 (14%) as high-grade lesions. The distribution of recurrent cases across histological grades did not suggest a consistent recurrence pattern in this cohort [19,20].

Most recurrent cases presented as mammographic microcalcifications (19/21, 90%), whereas only 2 lesions appeared as nodules on imaging. All recurrent cases were estrogen receptor–positive at the time of the initial DCIS diagnosis, and estrogen receptor positivity was confirmed in all recurrent tumors as well. No patients were known carriers of BRCA or other high-risk genetic mutations.

Among patients who developed recurrence, 20 of 21 (95%) had initially undergone breast-conserving surgery, reflecting the predominant use of conservative treatment in the study population, whereas only one patient had undergone mastectomy after declining the recommended radical surgical treatment. Eleven recurrent patients (52%) had not received adjuvant radiotherapy as part of the initial treatment strategy. None of the recurrent patients had received endocrine therapy. All recurrent cases showed negative surgical margins at initial treatment.

Most recurrences occurred between 12 and 60 months after treatment, with 8 recurrences observed between 12 and 48 months and 8 between 48 and 60 months. Only two recurrences occurred within the first year of follow-up. The clinical and pathological characteristics of patients with recurrence are summarized in Table 2.

**Figure 2:**
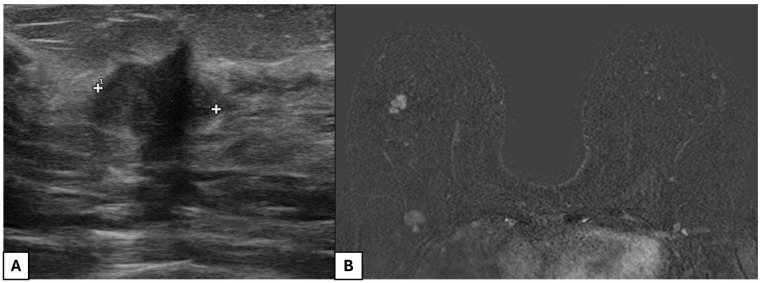
Ipsilateral invasive local recurrence at 59 months of follow-up in the same patient as Figure 1 (Case 1). (**A**) Ultrasound image showing a hypoechoic nodule with irregular margins detected at the surgical scar site, subsequently confirmed as invasive carcinoma on histopathology; “+” markers and the number “1” represent ultrasound measurement markers for lesion size assessment. (**B**) MRI showing mass-like enhancement consistent with recurrence.

**Figure 3:**
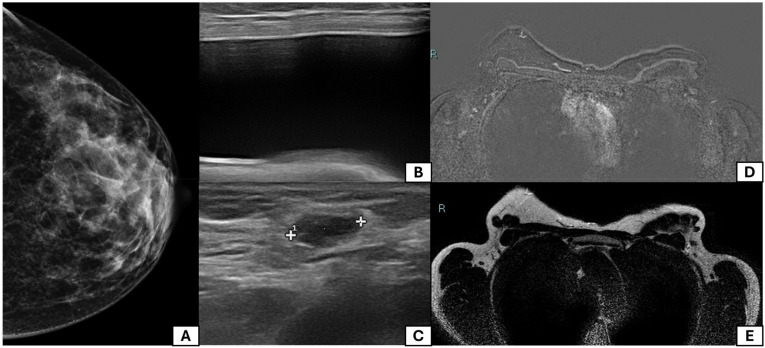
Lymph node recurrence at 54 months after nipple-sparing mastectomy in a 43-year-old patient with Grade 2 DCIS (Case 2). (**A**) Mammographic view showing pathological microcalcifications at diagnosis. (**B**) Post-operative ultrasound after nipple-sparing mastectomy with breast prosthesis placement. (**C**–**E**) Axillary ultrasound and MRI demonstrating the pathological lymph node in the axillary cavity; “+” markers and the number “1” represent ultrasound measurement markers for lesion size assessment.

Representative imaging findings are shown in Figure 1, Figure 2 and Figure 3. Figure 1 illustrates mammographic findings and post-operative follow-up in a patient with Grade 1 DCIS treated with breast-conserving surgery and radiotherapy. Figure 2 shows ipsilateral invasive local recurrence detected during follow-up by ultrasound and MRI. Figure 3 demonstrates axillary lymph node recurrence after nipple-sparing mastectomy detected on MRI follow-up.

## 4. Discussion

Ductal carcinoma in situ (DCIS) represents a significant proportion of screen-detected breast cancers and, although non-invasive by definition, carries a variable risk of local recurrence [1,2,3,4]. Our study confirms that, despite standard surgical treatment, only a minority of patients with pure DCIS experienced ipsilateral recurrence during radiological follow-up. In our cohort, 5% of patients developed recurrence, with a median time to recurrence of 42 months. Notably, more than half of the recurrences (57%) were non-invasive recurrent DCIS, supporting the concept that recurrence does not necessarily imply biological progression toward aggressive invasive disease [7,27].

Previous studies have reported similar recurrence rates, particularly after breast-conserving surgery without radiotherapy, which remains a debated management strategy. In our cohort, 52% of recurrent patients had not received adjuvant radiotherapy as part of the initial treatment approach. Although the exploratory design of our study does not allow definitive conclusions regarding treatment efficacy, this finding is consistent with previous literature suggesting a protective role of radiotherapy against ipsilateral recurrence [14,16]. At the same time, the growing interest in active surveillance and treatment de-escalation strategies for selected low-risk DCIS patients highlights the need for more accurate risk stratification tools [9,10,11].

Long-term data from prospective studies confirm that recurrence risk after excision without radiation increases over time, particularly beyond 15 years, underscoring the importance of extended follow-up [26,28].

An interesting observation in our cohort was that all recurrent cases were estrogen receptor–positive at the time of the initial DCIS diagnosis, whereas none of these patients had received endocrine therapy. Although endocrine treatment is not routinely indicated for all DCIS patients, its potential role in reducing recurrence risk in selected hormone receptor–positive patients remains an area of ongoing investigation [29]. The IBIS-II DCIS trial demonstrated comparable efficacy between anastrozole and tamoxifen in reducing locoregional and contralateral breast cancer events in postmenopausal women with hormone receptor–positive DCIS [29]. In our center, endocrine therapy is offered as part of a multidisciplinary decision-making process in selected ER-positive DCIS patients according to current guidelines. The distribution of recurrent cases across low-, intermediate-, and high-grade lesions did not suggest a consistent recurrence pattern in this cohort. These findings support the growing evidence that histological grade alone may be insufficient to reliably predict recurrence behavior in DCIS [19,20]. However, the relatively limited number of recurrence events reduces the statistical power of exploratory subgroup analyses, and the absence of statistically significant associations should not be interpreted as evidence of the absence of association.

Additionally, no distant metastases or disease-related deaths were observed during follow-up, further confirming the excellent overall prognosis of pure DCIS reported in the literature [7,18]. Importantly, even the patient who experienced lymph node recurrence after mastectomy did not show subsequent systemic disease progression during follow-up.

Younger age appeared more frequently among recurrent patients in our cohort, in line with previous reports suggesting that younger women may have a higher recurrence risk [19,30]. Nevertheless, the retrospective design and limited number of recurrence events preclude definitive conclusions regarding the independent role of age as a prognostic factor.

From a surgical perspective, most recurrences occurred in patients initially treated with breast-conserving surgery, reflecting the predominant use of conservative treatment within the study population. Therefore, our findings should not be interpreted as demonstrating equivalence between breast-conserving surgery and mastectomy in terms of recurrence risk [17,18]. Treatment allocation was not randomized and reflected multidisciplinary clinical decision-making, patient preference, age, comorbidities, margin status, and evolving institutional practice over the study period. Consequently, potential confounding factors may have influenced recurrence patterns and treatment distribution.

Overall, our findings support the need for more refined and individualized approaches to DCIS management. Traditional clinicopathological variables alone may be insufficient to consistently identify patients at higher risk of recurrence. Future research integrating molecular profiling, imaging phenotypes, and patient-specific clinical factors may help improve risk stratification and reduce overtreatment in selected patients with DCIS [31]. Importantly, the absence of statistically significant associations in this cohort should be interpreted with caution. The relatively small number of ipsilateral recurrence events limits statistical power and may preclude detection of modest but clinically meaningful associations. This study has several limitations. First, its retrospective single-center design may introduce selection bias. Contralateral recurrences were excluded from the analysis as they likely represent biologically independent tumors rather than true recurrences of the index DCIS, and their inclusion would have introduced a confounding factor in the evaluation of ipsilateral recurrence patterns. Second, the relatively small number of recurrence events limited the possibility of performing robust multivariable or time-to-event analyses. Third, treatment allocation was not randomized and may have introduced additional confounding factors, including multidisciplinary clinical decision-making, patient preference, age, comorbidities, and evolving institutional practice over the study period. Finally, although the follow-up duration was sufficient to detect early recurrence events, longer follow-up is needed to better assess long-term outcomes and late recurrences.

## 5. Conclusions

Our findings confirm that recurrence after treatment for pure DCIS is relatively uncommon and often non-invasive, in line with previous large population-based studies [7,18]. Histological grade and tumor size were not significantly associated with recurrence in our cohort; however, this finding should be interpreted cautiously, given the limited number of recurrence events and the reduced statistical power of the analysis, as the absence of statistical significance should not be interpreted as the absence of association. These results suggest that traditional clinicopathological variables appeared insufficient to consistently identify recurrence patterns, supporting the need for more refined risk stratification approaches integrating molecular profiling and imaging biomarkers [19,20]. Although most recurrences occurred after breast-conserving surgery, this finding likely reflects the predominance of BCS in our cohort and should not be interpreted as evidence of inferior oncological outcomes compared to mastectomy. The role of radical surgery in selected low-risk cases deserves further prospective investigation [17,18]. Further prospective studies with molecular profiling are needed to refine risk stratification and guide de-escalation strategies [30,32].

## Figures and Tables

**Table 1:** Clinical and pathological characteristics of the overall study cohort.

Variable	Overall Cohort
Patients	403
Pure DCIS cases	417
Mean age at diagnosis	56.7 ± 10.2 years
Low-grade (DIN1)	75 (18%)
Intermediate-grade (DIN2)	246 (59%)
High-grade (DIN3)	96 (23%)
BCS only	162
BCS + RT	185
BCS + RT + ET	16
Masetctomy	28
Mean lesion size	16.0 ± 11.5 mm
Microcalcifications	394
Nodules	23
ER-positive	311 (75%)
Negative margins	414 (99.3%)
Necrosis absent	197 (47%)
Necrosis present	220 (53%)

**Table 2:** Clinical, pathological, and treatment characteristics of patients with ipsilateral recurrence.

Variable	Recurrence Group (*n* = 21)
Non-Invasive recurrence	12 (57%)
Invasive recurrence	8 (38%)
Lymph Node recurrence	1 (5%)
Median Age	57 years
Median time to recurrence	42 months
Low-grade DCIS	5 (24%)
Intermediate-grade DCIS	13 (62%)
High-grade DCIS	3 (14%)
Mean lesion size	12.2 ± 9.2 mm
Microcalcifications	19 (90%)
Nodules	2 (10%)
BCS only	10
BCS + RT	10
Mastectomy	1
No RT	11 (52%)
Endocrine therapy	0
Negative margins	21 (100%)

## Data Availability

The data presented in this study are available on request from the corresponding author due to privacy and ethical restrictions.
